# Genomic and Immune Profiling of Esophageal Squamous Cell Carcinoma Undergoing Neoadjuvant Therapy Versus Upfront Surgery Identifies Novel Immunogenic Cell Death‐Based Signatures for Predicting Clinical Outcomes

**DOI:** 10.1002/mco2.70171

**Published:** 2025-04-02

**Authors:** Peidong Song, Wenze Tian, Yujia Zheng, Sukai Xu, Zihao Hu, Xing Jin, Xuejuan Zhu, Lijie Tan, Donglai Chen, Yongbing Chen

**Affiliations:** ^1^ Department of Thoracic Surgery the Second Affiliated Hospital of Soochow University Suzhou China; ^2^ Department of Thoracic Surgery the Affiliated Huai'an First People's Hospital of Nanjing Medical University Huai'an China; ^3^ Department of Thoracic Surgery Zhongshan Hospital Fudan University Shanghai China

**Keywords:** esophageal squamous cell carcinoma, genomic landscape, immunogenic cell death, neoadjuvant therapy, tumor microenvironment

## Abstract

It remains undetermined regarding the impact of neoadjuvant therapy on immunogenic cell death (ICD) and subsequent tumor microenvironment (TME) remodeling in esophageal squamous cell carcinoma (ESCC). And it is of paramount significance to identify beneficiaries from neoadjuvant therapy in treatment‐naïve ESCC. In this study, 88 ESCC samples undergoing neoadjuvant therapy plus surgery (NA+S) or surgery alone (SA) were subjected to bulk‐RNA sequencing. A five‐gene RINscore incorporating ICD‐related signature genes with TME‐based hub genes was established to predict clinical outcomes and pharmacological responses, in which SLAMF7 and IL1R1 were selected out as co‐expressed genes. The regulatory mechanism of the repressive co‐transcription factor BATF of SLAMF7 and IL1R1 was further demonstrated. Our data demonstrated that NA+S led to high abundance in kinds of T helper cells, nature killer T cells and M1‐like macrophages with increased CD8+T cells infiltration compared with SA. ICD phenotypes were further characterized in treatment‐naïve ESCC to determine their differences in TME and potential benefits from NA. Our findings not only offered novel insights into the distinct TME and ICD profiles of ESCC undergoing different therapeutic modes, but also provided the RINscore, which may aid oncologists in determining individualized (neo)adjuvant immunotherapy regimen.

## Graphical abstract

A total of 88 ESCC samples undergoing neoadjuvant therapy plus surgery (NA+S) or surgery alone (SA) were obtained and subjected to bulk RNA sequencing. An external cohort consisting of 95 ESCC undergoing SA was retrieved from The Cancer Genomic Atlas as validation. A five‐gene RINscore incorporating ICD‐related signature genes with TME‐based hub genes was established, with pivotal genes and their co‐transcriptional mechanisms elucidated in vitro.

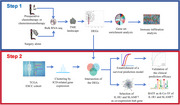

## 1 Introduction

Esophageal cancer is one of the commonest gastrointestinal malignancies [1]. More than 90% of ECs in China are categorized into esophageal squamous cell carcinoma (ESCC) in histology, which are characterized with an insidious onset, aggressive behaviors, and poor prognosis [2, 3, 4]. Hitherto, several multicenter prospective clinical trials have identified the superiority of neoadjuvant therapy plus surgery (NA+S) in the treatment of locally advanced ESCC, irrespective of neoadjuvant therapy regimens [5, 6, 7]. However, due to concerns over financial burden or physical conditions, a minority of patients are likely to display poor compliance with neoadjuvant therapy. In addition, there is no denying that many ESCC patients could not derive any benefit from neoadjuvant therapy due to complex heterogeneity in tumor microenvironment (TME), especially in the context of increasing use of immune checkpoint inhibitors (ICIs) [8]. Therefore, it is of paramount significance to identify beneficiaries among whom neoadjuvant therapy can prolong survival.

The TME of ESCC is usually featured with massive expression of inhibitory molecules from tumor cells as well as remarkable infiltration of immunosuppressive cells, such as exhausted CD4+, CD8+T cells, NK cells, and myeloid‐derived suppressor cells (MDSCs) [9, 10]. A recent study has demonstrated that neoadjuvant therapy enhances the activation of immune cells, thereby potentially improving the efficacy of anti‐PD‐L1 therapy [11]. Therefore, investigating the mechanisms by which neoadjuvant therapy recruits and attracts immune cell infiltration, as well as its impact on sensitivity/resistance to immunotherapy, remains a meaningful pursuit.

Regulated cell death (RCD), a form of cell death, has been recognized as a critical process in immunomodulation [12, 13]. Immunogenic cell death (ICD), one of the special forms of RCD, is characterized by the release of plentiful damage‐associated molecular patterns (DAMPs), including calreticulin (CALR), high‐mobility group proteins 1 (HMGB1), heat shock proteins (HSP70, HSP90), and so on [14]. DAMPs can activate dendritic cells (DCs) and cytotoxic T lymphocytes (CTLs) to establish long‐term adaptive immunity and can also mediate tumor immune tolerance by recruiting MDSCs [15]. Hitherto, several studies have confirmed that ICD can organize the TME [16, 17]. Notably, it has been recognized that neoadjuvant therapy might induce ICD by enhancing antigenicity of tumor cells [14], as neoadjuvant chemotherapeutics or radiation commonly makes tumor‐associated (neo)antigens exposed under pressure [18]. However, it remains undetermined regarding the impact of neoadjuvant therapy on ICD and subsequent TME remodeling in ESCC.

In this study, we aimed to characterize the distinct TME landscapes of ESCC patients receiving different treatment modes by profiling 88 ESCC specimens undergoing NA+S or surgery alone (SA). Besides, we also established a survival prediction model incorporating ICD‐related signature genes with TME‐based hub genes which might aid in identifying beneficiaries from NA. Ultimately, we sought to investigate the biological function and underlying regulatory mechanisms of the selected ICD‐related hub genes using in vitro experiments.

## 2 Results

### 2.1 Delineation of Genomic Landscapes Between NA+S Versus SA

**FIGURE 1:**
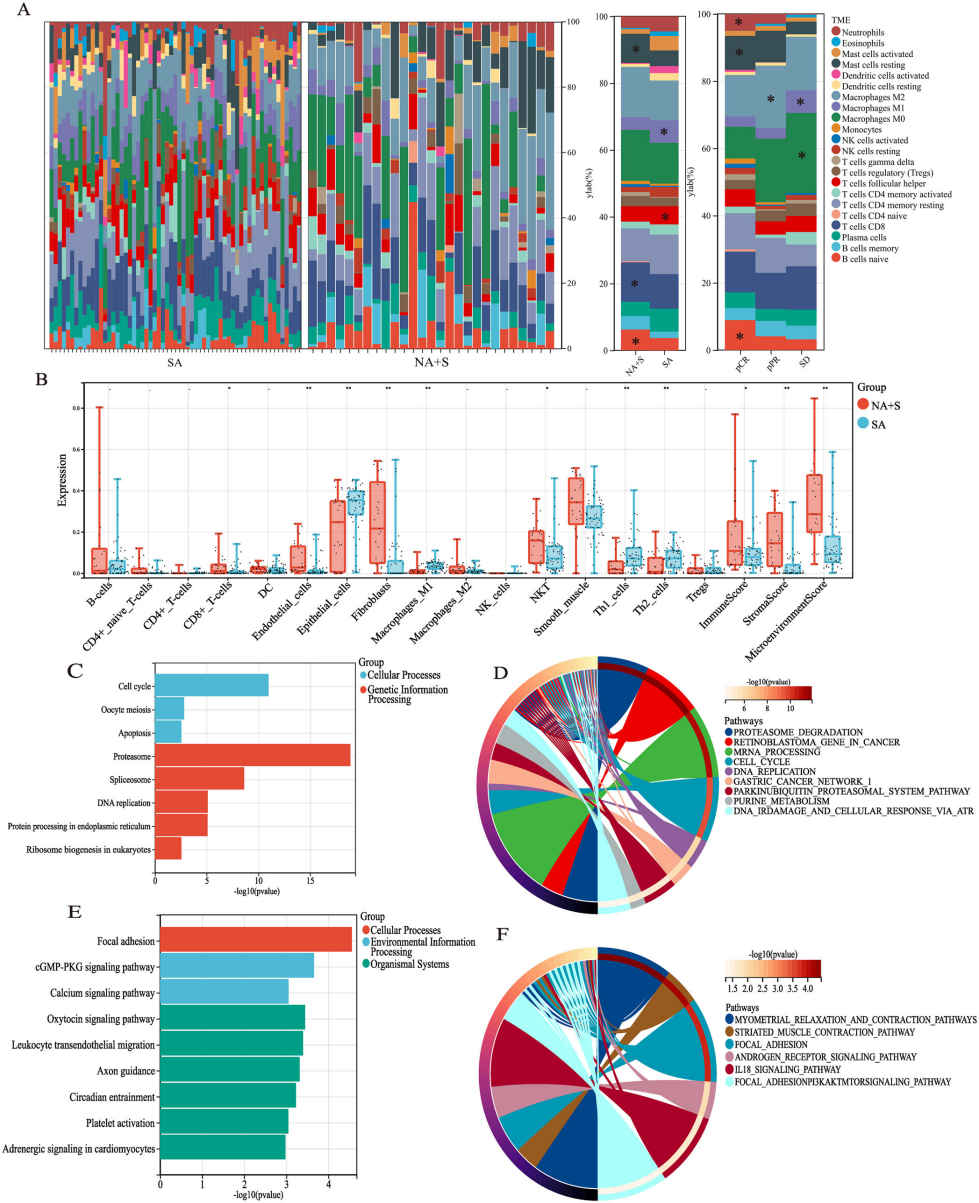
Differences in genomic and immune profiles of the ESCC samples between NA+S and SA groups. (A) Quantification of immunocytes in treatment‐experienced and ‐naïve lesions; (B) Boxplot of differences in TME compositions between different treatment modes; (C) KEGG and (D) Wiki pathways analysis of downregulated gene enrichment between the two groups; (E) KEGG and (F) Wiki pathways analysis of upregulated gene enrichment between the two groups. NA+S = neoadjuvant plus surgery; SA = surgery alone. ‐*p *> 0.05, *p < 0.05, **p < 0.01.

The demographic characteristics of our primary cohort were summarized in Table 1. After dimensionality reduction by PCA in whole transcriptome data, the transcriptomic data of four samples in the NA+S group and five samples in the SA group were discarded (Figure S1). To xCell analysis showed that compared with treatment‐naïve lesions, treatment‐experienced lesions were characterized by distinct immune and stromal cell infiltration atlas including CD8+T cell, helper T cell, cancer‐associated fibroblasts (CAFs), and so on (Figure 1A,B). In contrast, there was remarkable decreased infiltration of epithelial cells and M1‐like macrophages in the ESCC undergoing NA+S. Beyond that, in the NA+S group, increased infiltration of naive B cells, eosinophils, and neutrophils was observed in CR lesions compared with PR and SD ones. Besides, the abundance of M0‐ and M1‐like macrophages in the SD group outnumbered that of the CR and PR groups (Figure 1A,B). Our subgroup analysis also revealed that ESCC with a moderate to low grade in pathology following NA exhibited increased abundance of CAFs and higher stromal cell scores ((Figure S2). In addition, subgroup analysis by pathological Stages III and IV indicated that there was significantly higher infiltration of immune cells such as CD8+T cells and NKT cells in the NA+S group compared with the SA group (Figure S2). In particular, ESCC with advanced pathological stages (III and IV) exhibited a higher abundance of DC cells and M1‐like macrophages compared to Stages I and II (Figure S3).

**TABLE 1:** Patient demographic characteristics.

	NA+S (*n* = 27) No. (%)	SA (*n* = 61) No. (%)	Total (*n* = 88)	*p*
Gender				0.36
Male	19 (21.59%)	35 (39.77%)	54 (61.36%)	
Female	8 (9.09%)	26 (29.55%)	34 (38.64%)	
Age (year)				
Mean ± SD	66.59±8.02	68.08±6.93	67.63±7.27	
Median (min‐max)	67 (46, 78)	69 (54, 86)	69 (46, 86)	
Pathological stage (yp/p)				< 0.001
I (including ypT0N0, pCR)	18 (20.45%)	2 (2.27%)	20 (22.73%)	
II	3 (3.41%)	30 (34.09%)	33 (37.50%)	
III	6 (6.82%)	23 (26.14%)	29 (32.95%)	
IV	0	6 (6.82%)	6 (6.82%)	
Lymph nodes involvement				0.80
N0	14 (15.91%)	35 (39.77%)	49 (55.68%)	
N+(1∼any)	13 (14.77%)	26 (29.55%)	39 (44.32%)	
Grade				1.00
3	11 (12.50%)	24 (27.27%)	35 (39.77%)	
1–2	16 (18.18%)	37 (42.05%)	53 (60.23%)	
Tumor location				0.80
Upper‐middle	13 (14.77%)	26 (29.55%)	39 (44.32%)	
Lower	14 (15.91%)	35 (39.77%)	49 (55.68%)	
RECIST				NaN
Complete response	13 (48.15%)	—	—	
Partial response	9 (33.33%)	—	—	
Stable disease	5 (18.52%)	—	—	

*Note*: The pathological staging of this study was based on the 8th edition of the ESCC‐TNM staging criteria issued by the Joint American Council on Cancer/International Union for Cancer Control (AJCC/UICC).

Abbreviations: NA+S = neoadjuvant plus surgery; RECIST = response evaluation criteria in solid tumor; SA = surgery alone.

A total of 1026 DEGs (fold change > 8, adjust *p *< 0.01) between the NA+S and the SA groups were identified (Figure S4). Analysis of KEGG and Wiki revealed that 611 downregulated genes after NA were enriched in cellular processes, such as cell cycle and genetic information processing, including DNA repair, proteasome degradation, mRNA, and protein processing (Figure 1C,D). In addition, the majority of the upregulated genes after NA were found to be enriched in focal adhesion pathways and striated muscle contraction pathway (Figure 1E,F). The findings of the TME and gene enrichment analyses indicated that NA significantly altered the genomic landscape of ESCC.

### 2.2 Identification of ICD‐Associated Subtypes in Treatment‐Naïve ESCC

**FIGURE 2:**
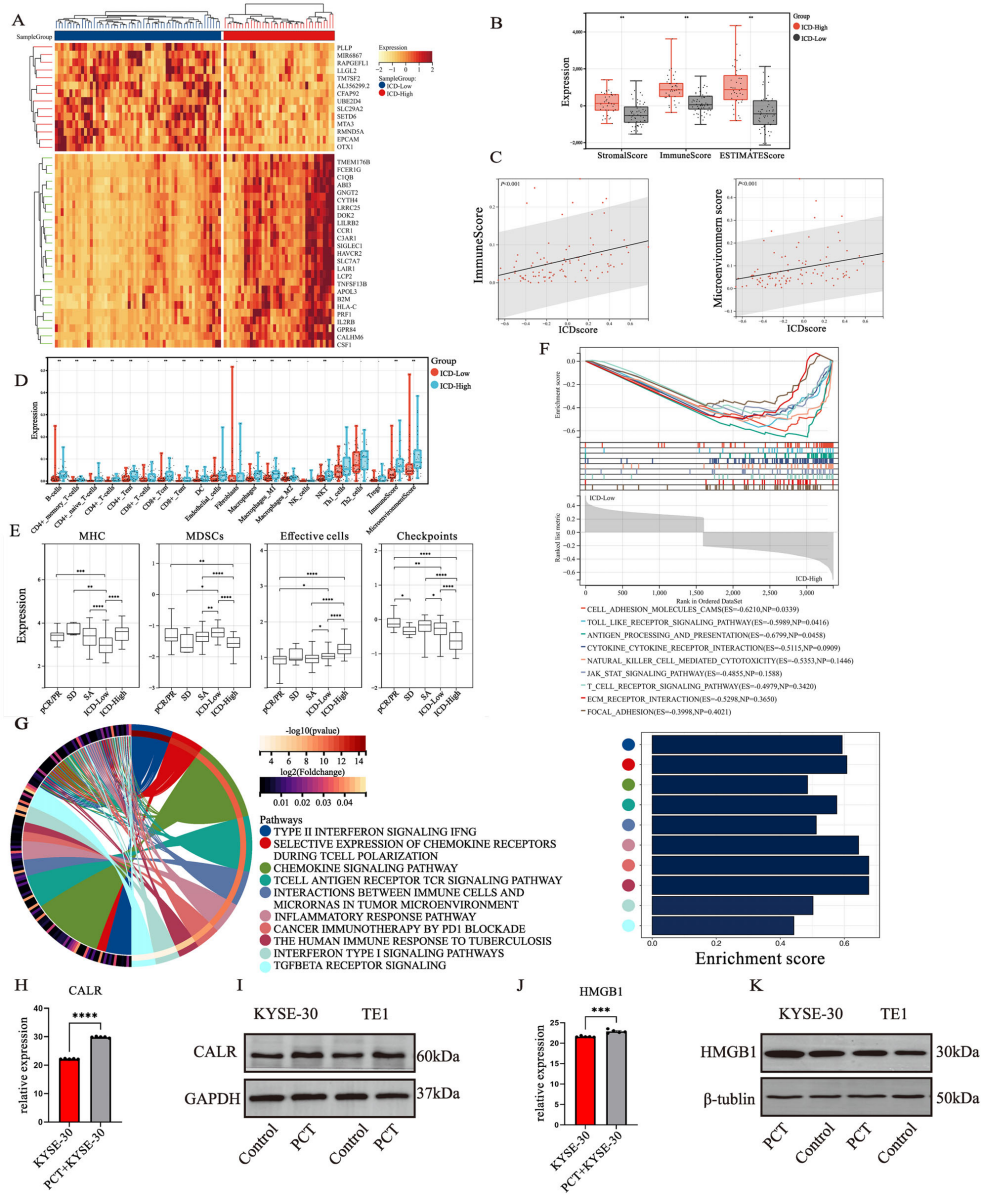
ICD clustering of ESCC samples in TCGA database and disparate TME between ICD^High^ and ICD^Low^. (A) Heatmap of the top 40 up‐ and downregulated DEGs; (B) Bar plot of ESTIMATE analysis between the High‐ and Low‐ICD clusters; (C) Positive correlation of ICD score and Immune score as well as microenvironment score; (D) Compared with the ICD^low^ group, the infiltration of most immune cells increased in the ICD^high^ group; (E) Boxplots of expression of MHC and immune checkpoints, MDSCs, Effective cells, in different subgroups; (F) KEGG enrichment mapping of intercluster DEGs; (G) Wiki pathways analysis showing the enrichment of multiple functional pathways in the ICD^high^ group; (H, I) Immunoblots for CALR protein expression of PC‐treated KYSE30 and PC‐treated TE‐1 cells. mRNA expression of CALR was determined by RT‐PCR; (J, K) Immunoblots for HMGB1 protein expression of PC‐treated KYSE30 and PC‐treated TE‐1 cells. mRNA expression of HMGB1 was determined by RT‐PCR. ICD = immunogenic cell death; MHC = major histocompatibility complex; MDSCs = myeloid‐derived suppressor cells; PCT = paclitaxel and cisplatin treated. ‐*p *> 0.05, *p < 0.05, **p < 0.01.

ICD‐related genes in ESCC patients undergoing SA from TCGA dataset (Table S1) were classified into two clusters via unsupervised clustering (Figure S5). The ICD score for each sample was calculated by ssGSEA according to 34 ICD‐related genes (Table S2), and ICD^high/low^ groups were defined by ICD score (Figure S6). A total of 227 upregulated genes and 728 downregulated genes were identified between the two groups (fold change > 5, adjust *p *< 0.01). The top 40 DEGs are shown in the heatmap (Figure 2A). It was suggested that the ICD score was positively correlated with the immune score and microenvironment score (Figure 2B‐C). Correspondingly, xCell analysis revealed that most immunocytes such as B cells, CD8+T cells, and macrophages displayed higher infiltration levels in the ICD^high^ group than in the ICD^low^ group (*p *< 0.001) (Figure 2D). Furthermore, the ICD^high^ group showed lower MDSC infiltration levels but elevated MHC expression and effector cell infiltration levels compared with the ICD^low^ group (Figure 2E). It was also identified that the ICD^high^ group was characterized by activation of tumorigenesis‐related pathways in addition to immunoregulatory pathways, such as interferon Type I/II signaling pathway and transforming growth factor‐beta (TGF‐β) pathway (Figure 2F,G). In our experiments in vitro, it was observed that the expression of two DAMPs genes, CALR and HMGB1, was upregulated under the stimulation of paclitaxel and cis‐platinum in KYSE‐30/TE‐1 cells compared to that in the control group (Figure 2H–K). These results demonstrated that the ICD^high^ group presented a more active TME. Moreover, paclitaxel and cisplatin could act as ICD inducer in ESCC.

### 2.3 Development and Validation of RINscore to Predict Clinical Outcomes and Drug Sensitivity

**FIGURE 3:**
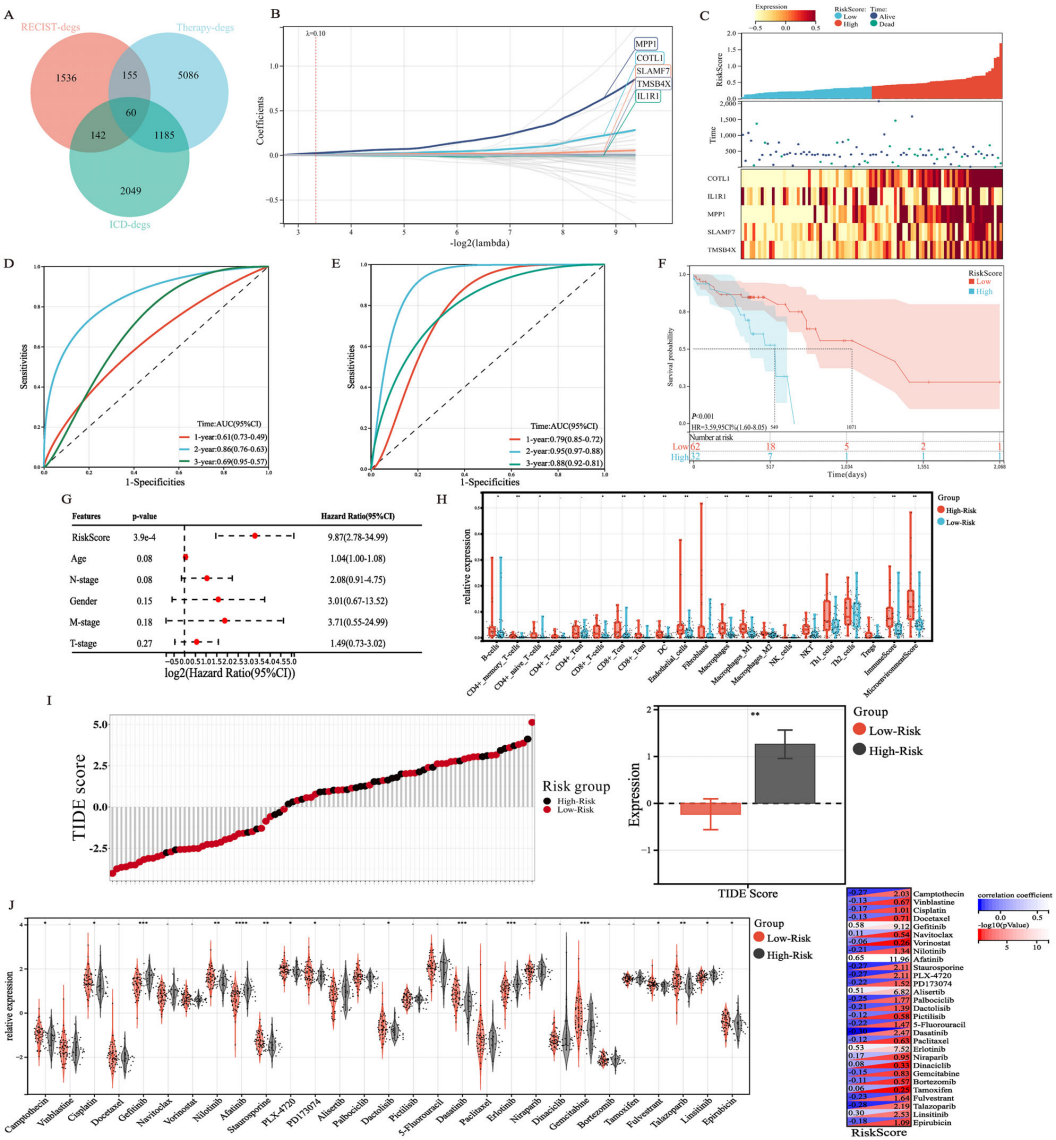
Development and validation of a five‐gene signature to predict clinical outcomes. (A) Venn diagram shows the intersection of communal genes of the three DEG sets; (B) Lasso–Cox regression analysis identified five signature genes; (C) Visualization of risk score distribution and survival time corresponding to the expression levels of the five prognostic genes; (D) The ROC curve of the five‐gene signature indicated that the model displayed satisfactory predictive ability at 1‐, 2‐, and 3‐year time points; (E) The five‐gene signature's predictive ability at 1‐, 2‐, and 3‐year time points in N0 subgroup; (F) Kaplan–Meier analysis revealed that the five‐gene signature could distinguish overall survival in TCGA cohort; (G) Multivariate analysis confirmed the risk score as an independent predictor of survival; (H) With the increase of risk coefficient, the infiltration of various immune cells increased; (I) The high‐risk group had a higher TIDE score and worse response to immunotherapy than the low‐risk group; (J) Predictive results of drug susceptibility between different risk groups. ‐*p *> 0.05, *p < 0.05, **p < 0.01.

It has been known that chemotherapeutics might be associated with ICD phenotypes [14]. Sixty candidate genes were obtained from the intersection of the two gene sets (TME‐based hub genes; DEGs of ICD^high^ vs. ICD^low^) for further analyses (Figure 3A). LASSO–Cox regression analysis was initially performed to screen 60 candidate genes, with five survival‐related signature genes selected out, including MPP1, COTL1, SLAMF7, TMSB4X, and IL1R1 (Figure 3B). A risk model based on the five signature genes were then developed to predict patient survival, entitled as RINscore (pCR/PR vs. SD; ICD^high^ vs. ICD^low^; NA+S vs. SA). Figure 3C illustrates the relationships among expression levels of the five signature genes and the RINscore as well as patient status. ROC analysis confirmed that the RINscore had good 1‐, 2‐, and 3‐year survival prediction accuracy (area under curve [AUC]: 1‐year OS = 0.61, 2‐year OS = 0.86, 3‐year OS = 0.69) (Figure 3D). Subgroup analysis by nodal metastasis revealed that the predictive ability of RINscore was better in the N0 group (AUC: 1‐year OS = 0.79, 2‐year OS = 0.96, 3‐year OS = 0.88) (Figure 3E) than N+ subgroup (AUC: 1‐year OS = 0.47, 2‐year OS = 0.79, 3‐year OS = 0.65) (Figure S7). Kaplan–Meier (KM) survival analysis demonstrated that the RINscore could also distinguish significantly different OS in TCGA‐ESCC cohort (HR = 3.59; 95CI%, 1.60–8.05; *p *< 0.001) (Figure 3F). Multivariate risk analysis further identified the RINscore as an independent prognosticator for ESCC (Figure 3G). Interestingly, it was revealed that B cells, CD4+T memory cells, dendric cells, macrophages, and CD8+T cells were more abundant in the high‐risk group (*p *< 0.05), accompanied with elevated overall immune score and microenvironment score (*p *< 0.05) (Figure 3H). Compared with the low‐risk group, the TIDE score was remarkably elevated in the high‐risk group, indicating poor responses to ICIs (Figure 3I). The oncopredict results suggested that the drug sensitivity of chemotherapeutics such as cisplatin elevated with increased RINscore (Figure 3J). The aforementioned results suggested that the RINscore possessed a strong predictive capacity for postoperative survival and responses to immunotherapy.

### 2.4 Correlation Between ICD and the Interaction of SLAMF7/IL1R1

**FIGURE 4:**
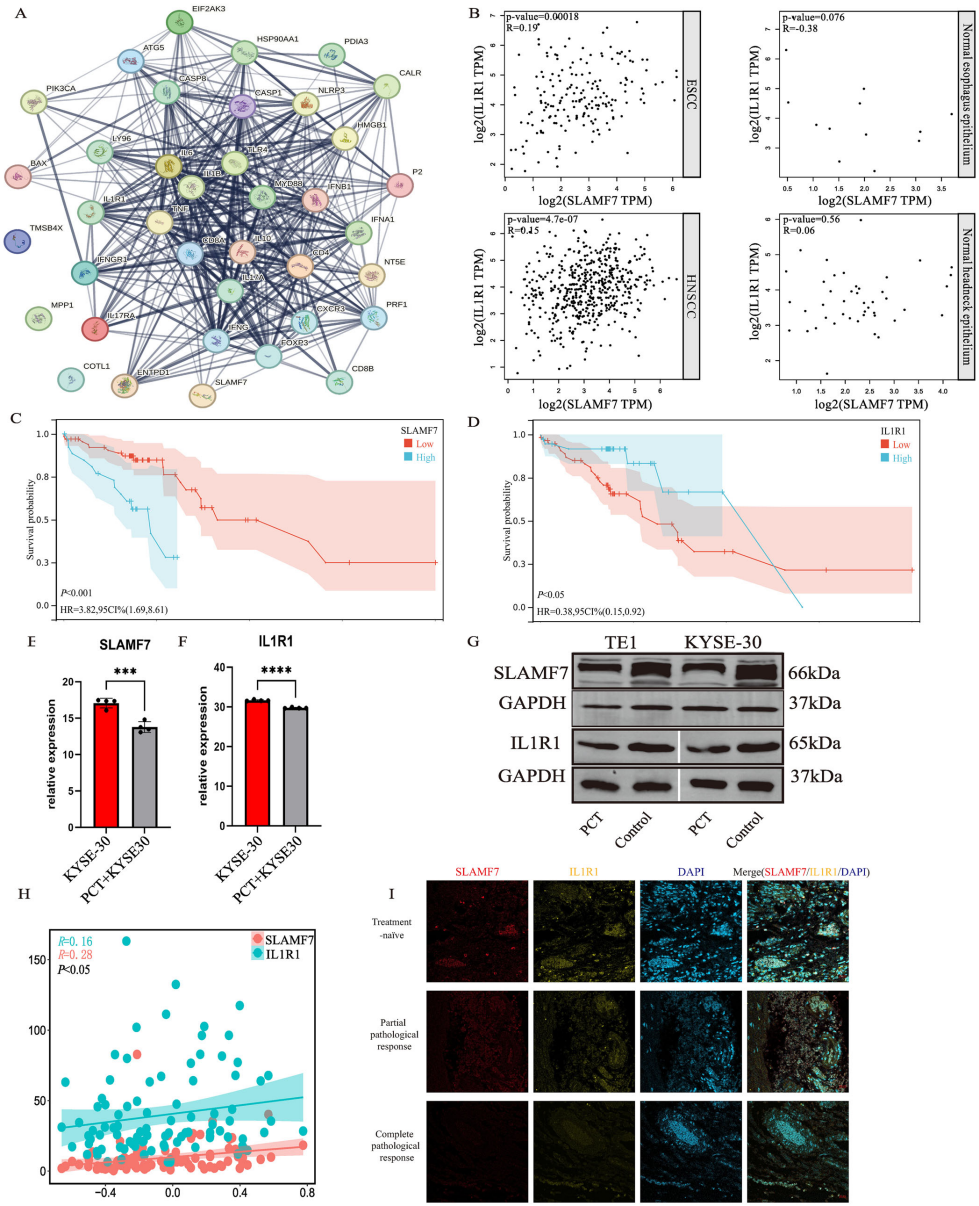
Correlation of SLAMF7 and IL1R1 interacting with ICD in ESCC. (A) STRING analysis showed that SLAMF7 and IL1R1 were involved in the ICD‐related genes co‐expression network; (B) Correlation of SLAMF7 and IL1R1 in ESCC/HNSCC and normal esophagus/head and neck tissues; (C, D) SLAMF7 was associated with shortened survival, while IL1R1 was the opposite; (E, F) mRNA expression of SLAMF7/IL1R1 was determined by RT‐PCR; (G) Immunoblots for SLAMF7/IL1R1 protein expression of PC‐treated KYSE30 and PC‐treated TE‐1 cells; (H) Both SLAMF7 and IL1R1 were considered to be relevant factors for ICD; (I) Representative immunofluorescence images of SLAMF7 and IL1R1 in ESCC specimens under different therapy mode and diverse pathological remission states. HNSCC = head and neck squamous cell carcinoma. Scale bar, 20 µm.

To delineate the underlying hub genes in RINscore and their association with ICD‐related genes, we performed STRING analysis to identify the interactions between five RINscore genes and 34 ICD‐related genes. STRING network analysis demonstrated that both IL1R1 and SLAMF7 from the RINscore were involved in the ICD‐related gene interaction network (Figure 4A). Therefore, IL1R1 and SLAMF7 were further investigated to link the ICD phenotype with their biological implications. Functional correlation analysis indicated that the expression level of SLAMF7 correlated with that of IL1R1 in ESCC, while the aforementioned relationship was not observed in normal esophageal epithelia. Similar trends were also seen in head and neck squamous cell carcinoma (HNSCC) (Figure 4B). KM analysis suggested that a higher SLAMF7 level was associated with poorer prognosis in ESCC. To the contrary, overexpression of IL1R1 was associated with better clinical outcomes (Figure 4C,D). Compared to the ESCC cells in the control group, the mRNA expression and protein levels of SLAMF and IL1R1 were reduced in PC‐treated KYSE‐30 and TE‐1 cell lines (Figure 4E–G). Both the expression levels of SLAMF7 and IL1R1 were identified to be closely associated with ICD (Figure 4H). The co‐expression trend of SLAMF7 and IL1R1 was observed by immunofluorescence staining for colocalization in ESCC sections. Notably, this trend became more pronounced with the increased extent of pathological remission (Figure 4I). In summary, SLAMF7 and IL1R1 acted as crucial prognostic factors in ESCC. NA led to decreased expression of SLAMF7 and IL1R1 in ESCC, and such an effect became more prominent along with the increase of pathological response rate.

### 2.5 Repressive Co‐Transcription Factor BATF of SLAMF7 and IL1R1 Promoter

**FIGURE 5:**
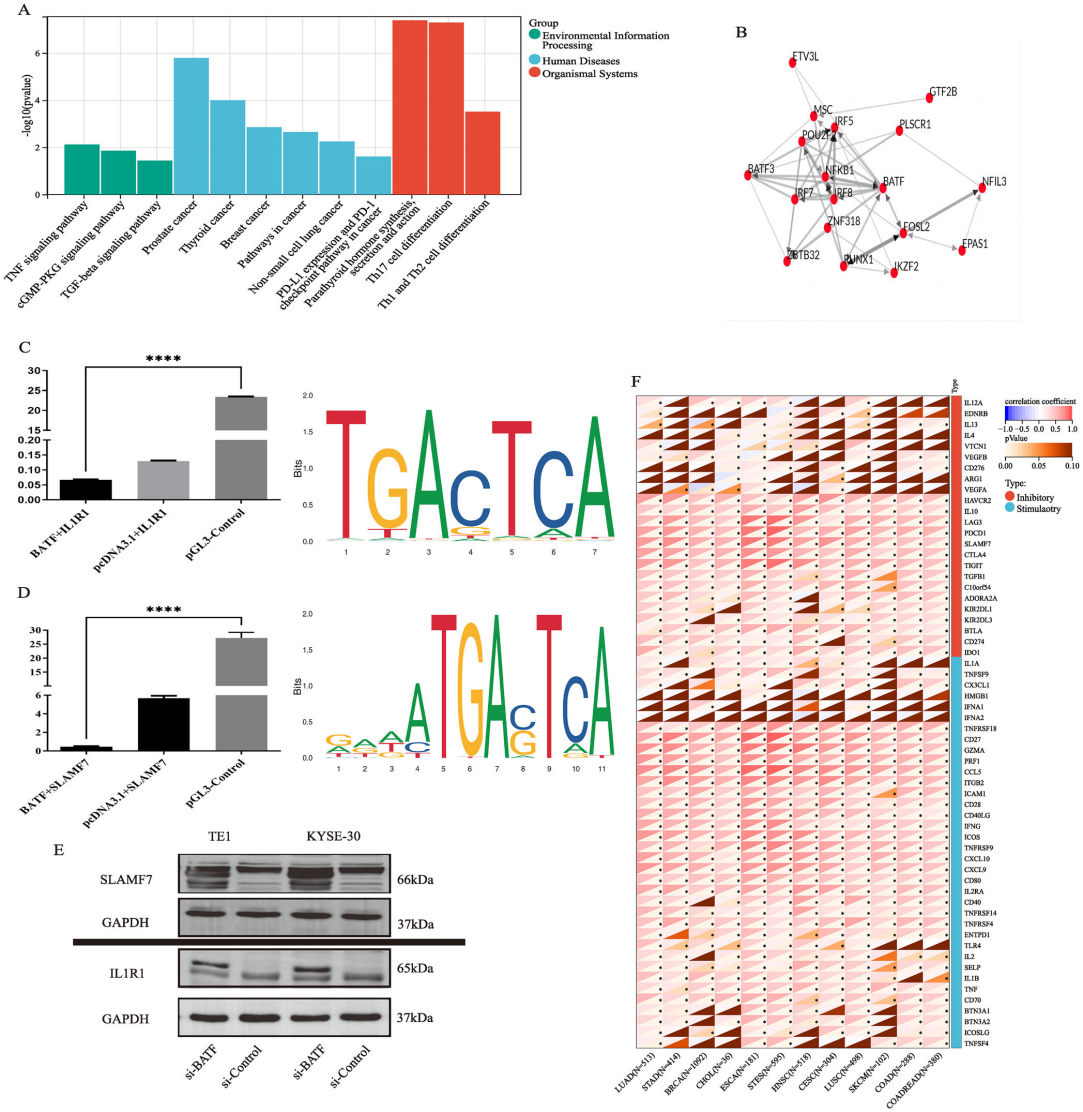
Promoter regulation of SLAMF7/IL1R1 by BATF. (A) Enrichment analysis of the top 20 Co‐TFs of SLAMF7 and IL1R1; (B) BATF was chosen as the hub TF for in vitro experiment validation. (C, D) Predicted results of dual‐luciferase reporter gene assay, and the binding sites of BATF and IL1R1/SLAMF7 gene promoters; (E) Immunoblots for SLAMF7/IL1R1 protein expression of sh‐BATF KYSE30 and sh‐BATF TE‐1 cells; (F) Correlation between the expression of BATF and 60 Immunomodulator genes.

To unravel the potential relationship between SLAMF7 and IL1R1, we analyzed the co‐transcription factors (Co‐TF) of the two genes. Co‐TF analysis showed that the top 20 candidate TFs of SLAMF7 and IL1R1 were enriched in the oncological pathways such as TNF/TGF‐β signaling pathway, PD‐1/L1 pathway, and so on (Figure 5A). The TF net analysis further identified BATF as the hub TF for subsequent validation in experiments in vitro (Figure 5B). Dual‐luciferase gene reporter assays demonstrated the inhibitory effect of BATF on IL1R1 (Figure 5C) and SLAMF7 promoters (Figure 5D). Figure 5C,D also illustrates the possible binding sites between BATF and IL1R1 promoters and those between BATF and SLAMF7 promoters. To determine the role of BATF in ESCC cells, we established BATF‐knockdown KYSE‐30 and TE‐1 cell lines. Western blot revealed that BATF knockdown significantly increased the expression levels of IL1R1 and SLAMF7 (Figure 5E). In addition, we also observed that the expression level of BATF was associated with a number of immunomodulatory genes in the pan‐cancer species obtained from TCGA dataset (Figure 5F). The experiments in vitro demonstrated that the high expression of BATF, as a co‐transcription factor of SLAMF7 and IL1R1, could significantly suppress the expression of SLAMF7 and IL1R1. In addition, BATF displayed potential immune regulatory ability across pan‐cancer.

## 3 Discussion

Along with NA+S becoming the standard treatment for locally advanced ESCC [19], there have been unceasing debates over the solutions to improving NA efficacy and the indications for prescribing adjuvant ICIs. The ideal NA regimens should not only kill cancer cells but also activate the immune system to eliminate any residual cancer cells surviving from induction treatment. Unlike apoptosis, RCD generates second messengers that act on immune cells in the TME, alerting them of danger [20]. Thus, chemotherapy‐driven ICD, a unique kind of RCD, has captured increasing attention. To our best knowledge, our study has been the first one to investigate the differences in TME landscape in ESCC undergoing NA+S and SA.

Several studies have shown that exhausted T cells, NK cells, regulatory T cells, M2‐type macrophages, and tolerant DCs dominate the TME of ESCC, forming an immunosuppressive context to compromise the efficacy of ICIs [21, 22]. Meanwhile, the infiltration of MDSCs and CST1+myofibroblasts also contributed to the organization of immunosuppressive TME of ESCC [23]. In our study, increased intratumoral infiltration of CD8+T cells was particularly observed after NA, which was also seen in the ICD^high^ group. Infiltration of CTLs might be attributed to the production of CCL5 [24], CXCL10 (Cys‐X‐Cys chemokine ligand 10) and CXCL9 induced by ICD [14, 20]. Therefore, ICD probably plays a crucial role in recruiting and activating CD8+T cell in ESCC after NA [25]. From our data, the ESCC samples in the ICD^low^ group tended to be described as an “immune desert” subtype due to scarce immunocytes and poor immunogenicity. In contrast, the ICD^high^ group could be deemed as an “immune inflamed” subtype, with abundant CTLs infiltration in addition to massive exposure of immune checkpoints [26, 27]. Nevertheless, after the RCD of tumor cells, the release of DAMPs and recruitment of MDSCs could also result in immune tolerance and reestablishment of immune escape [28]. Therefore, ICD could be also regarded as a double‐edge sword in ESCC. Adjuvant ICIs might be prescribed timely once the so‐called “immune inflamed” subtype of the primary tumor was identified [29].

The RINscore established in this study demonstrated a strong predictive capability for postoperative survival and responses to NA, outperforming a recent prediction model based on pan‐cell death for ESCC patients without lymph node metastasis [30]. However, our prediction model needs additional large‐sample sequencing data with long‐term survival information for validation and amelioration.

In terms of SLAMF7, a number of studies have investigated its role in innate and adaptive immunocytes [31, 32, 33, 34]. A recent study also identified high expression of SLAMF7 in exhausted T cells by identifying a subset of CXCL13+ CD8+T cells. Moreover, another study also reported that SLAMF7 and IL1R1 expressed on macrophages exhibited a synergistic antitumor effect, which might induce immune exhaustion after paclitaxel stimulation [35]. Hitherto, little has been known regarding the roles of SLAMF7 and IL1R1 in ESCC cells and their regulatory relationships. It was proposed by our in silico analysis that SLAMF7 and IL1R1 might be critical in mediating ICD in ESCC with BATF as their co‐transcription factor. Our experiments in vitro further uncovered the repressive role of BATF in regulating the transcription of SLAMF7 and IL1R1. More importantly, the co‐expression trend of SLAMF7 and IL1R1 remained steady in different lesions displaying distinct extent of pathological responses to NA.

Although our study provided a novel perspective on genomic and immune profiling for ESCC, there were still shortcomings. Undeniably, the number of ESCC samples after NA was limited, and the survival data have not been usable due to the short‐term surveillance after esophagectomy. Given that TCGA data were predominantly derived from individuals with European and American descent, it is imperative to conduct further validation to ascertain the generalizability of our findings to the Asian and the African populations. Furthermore, the accuracy of RINscore in predicting ICIs and chemotherapeutics has not been validated in real‐world cases.

Our findings not only offered provided novel insights into the distinct TME and ICD profiles of ESCC undergoing different therapeutic modes, but also identified RINscore as a predictor of clinical outcomes and pharmacological responses, which may aid oncologists in determining individualized (neo)adjuvant immunotherapy regimens.

## 4 Materials and Methods

### 4.1 Patient Selection and Sample Collection

A primary cohort of 88 ESCC patients was included whose fresh tissues were collected from the Second Affiliated Hospital of Soochow University during September 2020–December 2022. The exclusion criteria are listed below: (1) autoimmune diseases or other types of esophageal cancer (e.g., adenocarcinoma); (2) ESCC concurrent with other malignant tumors. All the patients underwent standard minimally invasive esophagectomy, among whom 27 received NA+S. The NA regimens included albumin‐bound paclitaxel + cisplatin (11 patients, 40.7%) and(or) albumin‐bound paclitaxel + platinum + pembrolizumab (16 patients, 59.3%), which were prescribed for two cycles. The second cycle of induction therapy was administered 3 weeks after the first treatment. All the patients underwent extended two‐fields lymphadenectomy [36]. R0 resection was achieved in all patients. The pathological staging was based on the eighth edition of the TNM staging system issued by the Joint American Council on Cancer/International Union for Cancer Control [37].

The selected tumor specimens were pathologically diagnosed ESCC, with a diameter of 0.5 cm excised, immediately rinsed with 0.9% normal saline, and soaked in RNA stabilization reagent followed by storage in liquid nitrogen. Pathologists provided reports on pathological responses reflected by tumor regression grade (TRG). The degrees of pathological response were graded as complete response (CR), partial response (PR), and stable disease (SD). The transcriptomic data of another 95 ESCC patients undergoing SA were obtained from The Cancer Genome Atlas (TCGA) dataset as an external cohort.

### 4.2 RNA‐Seq Library Construction and Data Process

Total RNA was isolated and purified using TRIzol reagent (Invitrogen, USA) following the manufacturer's procedure. Briefly, poly(A) RNA was purified from 1 µg total RNA using Dynabeads Oligo (dT)25‐61005 (Thermo Fisher, USA). Then the poly(A) RNA was fragmented using Magnesium RNA Fragmentation Module (NEB, USA). Then RNA fragments were reverse transcribed to create the cDNA by SuperScript II Reverse Transcriptase (Invitrogen, USA). The products were purified and enriched via PCR to generate the final library. After testing quality using an Agilent 2100 Bioanalyzer (Agilent, USA). The sequencing platform employed in this study was the illumina Novaseq 6000 (LC‐Bio Technology Co. Ltd., Hangzhou, China).

Fragments Per Kilobase of exon model per Million mapped fragments (FPKM) values were obtained using the HISAT2 (https://daehwankimlab.github.io/hisat2), where the fastq files were mapped to a human reference genome (Homo sapiens, GRCh38). To choose genes with accurate expression value, we removed genes whose FPKM was 0 in more than 20% samples prior to subsequent analyses [38].

To obtain the reduced dimensionality matrix and searching outlier samples, we performed principal component analysis (PCA) with the R package stats (version 3.6.0). Differentially expressed genes (DEGs) between the NA+S and the SA groups were analyzed using the limma package (version 3.40.6) [39].

### 4.3 Assessment of TME Profiles and Anti‐Tumor Drug Efficacy

The transcriptome data were analyzed according to the methods from the following database websites: (1) Cell type enrichment analysis (xCell) was performed using the website tool (https://xcell.ucsf.edu) to estimate the immune score and microenvironment score. The immune score was utilized to evaluate the level of immune cell infiltration, while the microenvironment score was to assess TME activity [40, 41]; (2) CIBERSORTx (https://cibersortx.stanford.edu) was performed to calculate the abundance of 22 kinds of immunocytes; (3) ESTIMATE (https://bioinformatics.mdanderson.org/estimate/index.html) was performed to acquire stroma and immune scores, which are calculated based on the abundance of immune and stromal cells in tumor tissues; (4) Immunophenotypic score (IPS) was determined using the website tool (https://tcia.at/home) by assessing the expression of major histocompatibility complex (MHC) molecules, effector cells, and MDSCs. Immune checkpoints including CD27, PDCD1LG2, CD274, HAVCR2, TIGIT, LAG3, and CTLA4. Effective cells include active/effector memory (Tem) CD4+T cells, active/Tem CD8+T cells; (5) The standardized pan‐cancer dataset was derived from the UCSC (https://xenabrowser.net/) TCGA+GTEx database, from which the Pearson correlation coefficients between the expression of BATF and 60 immunomodulator genes was calculated (24 inhibitory genes and 36 stimulatory genes) [42]; (6) Tumor immune dysfunction and exclusion (TIDE) (http://tide.dfci.harvard.edu) score was also employed to predict response to ICIs; (7) Anticancer drug efficacy prediction using R package “oncoPredict.”

To determine communal genes associated with administration of NA and pathological responses to NA, we initially collected the DEGs in our data from different treatment modalities (NA+S vs. SA) and degrees of pathological remission (pCR/PR vs. SD) as TME‐based hub genes.

### 4.4 Clustering of ICD‐Related Genes and Calculation of ICD Score

The ICD‐related genes included in our study were based on previous studies [43, 44], in addition to the gene list obtained from Genecards (https://www.genecards.org). Eventually, a total of 34 genes were included (Table S2). We calculated the enrichment score of each sample according to the ICD‐related gene set using the single sample gene set enrichment analysis (ssGSEA). The higher the ICD score was, the more pronounced were the expression of ICD‐related genes. Cluster analysis was performed using the R package ConsensusClusterPlus [45].

### 4.5 Gene Set Enrichment Analysis (GSEA)

Data were downloaded from the Molecular Signatures Database (MSigDB; http://www.gsea‐msigdb.org/gsea/downloads.jsp) [46, 47]. The analyses were accomplished using GSEA software (http://www.broadinstitute.org/gsea/index.jsp).

### 4.6 Identification of Prognostic Genes in Key Module

LASSO–Cox analysis was performed to screen for major survival‐related genes [48]. Survival status and gene expression data were integrated for regression analysis by the R software package glmnet. The parameters were configured as family = cox, nlambda = 100, alpha = 1, and nfolds = 10. The optimal cut‐off value was determined to be 0.4509. Finally, five genes identified as independent predictors of survival were involved in the model, and the risk score of each sample was calculated based on the following formula:

(1) $$\mathrm{Risk}\;\mathrm{score}=\sum_{i=1}^{n}\begin{array}{c} \left(\beta_{i}\times expression\left(\mathrm{gen}e_{i}\right)\right) \end{array}$$

The variable “*i*” in the formula represented a model gene, “expression(gene*_i_*)” denoted the expression level of gene “*i*,” while “*β_i_*” signified the corresponding coefficient of this gene in the lasso regression results.

### 4.7 Survival Analysis

KM analysis [49] was conducted for comparison of the overall survival (OS) utilizing the “survminer” and “survival” packages in R. The receiver operating characteristic software package (pROC, version 1.17.0.1) was used to obtain area under the curve (AUC) at 1‐, 2‐, and 3‐year time points. Multivariate Cox proportional regression model was employed to determine whether the risk score was an independent predictor of OS in ESCC.

### 4.8 Cell Culture

KYSE‐30 (BNCC, Henan, China) and TE‐1 (Cell Bank, Shanghai, China) cell lines were cultured in RPMI 1640 (10% fetal bovine serum and 1% penicillin/streptomycin) (Beyotime, Shanghai, China). All cell lines were mycoplasma negative.

### 4.9 ESCC Cell Lines Treated With Paclitaxel and Cisplatin (PC)

KYSE‐30 and TE‐1 cells were continuously treated with gradually increased doses of paclitaxel and cisplatin (33069‐62‐4, 15663‐27‐1, MCE, USA). Briefly, cells were treated with 0.6 µmoL/mL paclitaxel and 4 µmoL/mL cisplatin in the 6‐well plates, and stable IC50 values were obtained after 48 h. The RNA and proteins used in the subsequent experiments were taken from cells after 48‐h culture.

### 4.10 Western Blot

Protein extraction was conducted by RIPA buffer (NCM biotech, China) containing PMSF (10:1) as previously described [50]. The antibodies used in the experiments are listed in Table S3.

### 4.11 Quantitative Real‐Time PCR

Primers (Table S4) were bought from Sangon biotech institute (Shanghai, China). The Prime‐Script RT Reagent Kit with gDNA Eraser (TaKaRa, D6110A) was used to reverse‐transcribe mRNA into cDNA. RT‐PCR was performed using SybrGreen on the AriaMX (Agilent).

### 4.12 Immunofluorescence Staining Assay

As previously described [50], ESCC sections were incubated with primary antibodies and secondary antibodies which prompted binding of different luciferins by tyramide signal amplification (TSA). DAPI was utilized for visualizing the cell nucleus. Images were scanned by fluorescence microscopes (ZEISS, Germany).

### 4.13 Co‐Transcription Factor Prediction and Dual‐Luciferase Reporter Gene Assay

In brief, ChIP‐X enrichment analysis version 3 (ChEA3 (maayanlab.cloud)) was used to predict co‐transcription gene. Jaspar analysis (https://jaspar.elixir.no/) was used to predict the binding site of a transcription factor to a promoter. The BATF binding sites in the promoters of wild‐type (WT) SLAMF7 and IL1R1 were designed and cloned into the luciferase vectors (Sangon biotech, Shanghai, China). After being cultured for 48 h, the cells were subjected to dual‐luciferase reporter gene assay kit (Sangon biotech, Shanghai, China) for assessing dual luciferase activities.

### 4.14 Transfection of siRNA

BATF siRNA and siRNA‐control was transfected into cells with Lipofectamine 2000 (Hanbio Biotechnology Co. Ltd., Shanghai, China). The detailed methods referred to our previous study [51]. Control siRNA and BATF siRNA were obtained by MedChemExpress, Shanghai, China.

### 4.15 Statistical Analysis

All statistical analyses were carried out using R (v.4.3.1) or GraphPad Prism software (V 9.0.0). Data were presented as mean ± standard error of the mean (SEM). For comparing multiple groups, an unpaired two‐tailed Student's *t*‐test was used. Statistical significance was suggested as: *p < 0.05, **p < 0.01, ns/‐: no significance.

## Author Contributions

**P.S**.: concept and study design, methodology, data curation, analysis and interpretation of data, manuscript writing. **W.T**.: methodology, manuscript writing, funding acquisition. **Y.Z**.: methodology, data curation, manuscript writing; **S.X**.: methodology. **Z.H**.: methodology, data curation. **X.J**.: data curation, funding acquisition. **X.Z**.: validation, funding acquisition. **L.T**.: validation. **D.C**.: concept and study design, writing – review and editing. **Y.C**.: writing – review and editing, funding acquisition. All authors have read and approved the final manuscript.

## Ethics Statement

This study was approved by the Institute of the Second Affiliated Hospital of Soochow University (EC–2024‐118). Informed consent was obtained from all participants in the study.

## Conflicts of Interest

The authors declare no conflicts of interest.

## Supporting information



Supporting information

## Data Availability

The data presented in this study are available in the article. The datasets used or analyzed during the current study are available from the corresponding author upon reasonable request.
