# Supplementary material for: Genomic and Immune Profiling of Esophageal Squamous Cell Carcinoma Undergoing Neoadjuvant Therapy Versus Upfront Surgery Identifies Novel Immunogenic Cell Death‐Based Signatures for Predicting Clinical Outcomes
Source: MedComm (2020). 2025 Apr 2;6(4):e70171. doi: 10.1002/mco2.70171 (PMC11965704; doi:10.1002/mco2.70171)

**Genomic and Immune Profiling of Esophageal Squamous Cell Carcinoma Undergoing Neoadjuvant Therapy Versus Upfront Surgery Identifies Novel Immunogenic Cell Death-Based Signatures for Predicting Clinical Outcomes**

Peidong Song, MD^#a^; Wenze Tian, MD^#c^; Yujia Zheng, MD^#a^; Sukai Xu, MD^a^; Zihao Hu, MD^a^; Xing Jin, MD^a^; Xuejuan Zhu, BS^a^; Lijie Tan, MD^*b^; Donglai Chen, MD, PhD^*b^; Yongbing Chen, MD, PhD^*a^

Affiliations:

a.Department of Thoracic Surgery, the Second Affiliated Hospital of Soochow University, Suzhou 215004, China

b.Department of Thoracic Surgery, Zhongshan Hospital, Fudan University, Shanghai 200032, China

c.Department of Thoracic Surgery, the Affiliated Huai’an First People’s Hospital of Nanjing Medical University, Huai’an 223300, China

#Drs. Song P., Tian W. and Zheng Y. equally contributed to the work.

*Drs. Tan L., Chen D. and Chen Y. were listed as co-senior authors.

*Correspondence to Dr. Yongbing Chen, Email: [chentongt@sina.com](mailto:chentongt@sina.com), Institutional Email: [ybchen@suda.edu.cn](mailto:ybchen@suda.edu.cn).


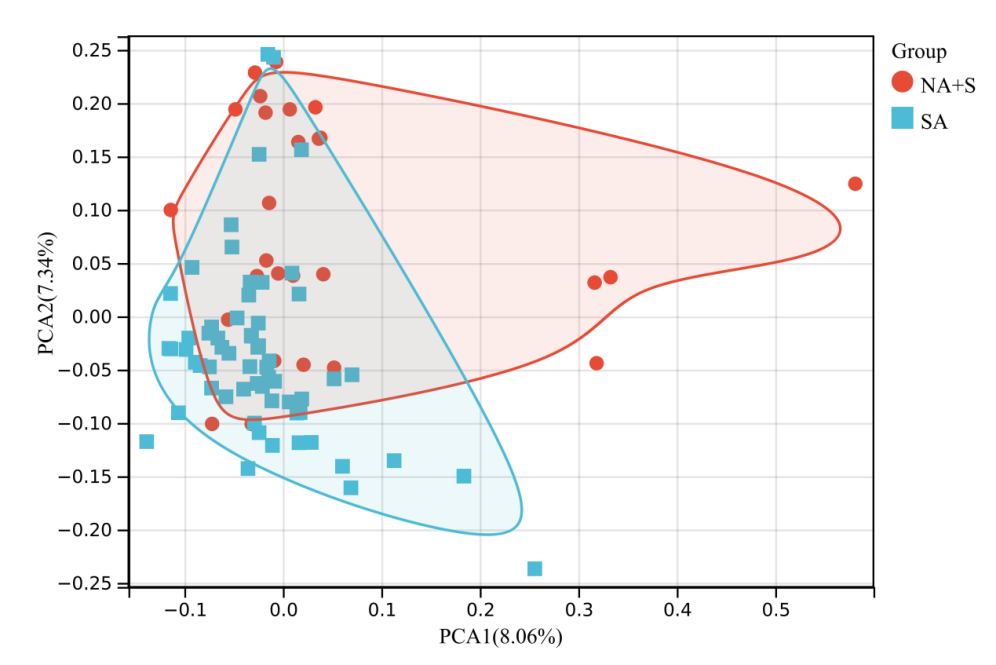


**Figure S1.** PCA in whole transcriptome for dimensionality reduction and sample exclusion.

**Figure S2.** Subgroup analyses by gender, grade of differentiation, and pathological stage in the NA+S group versus the SA group.

**Figure S3.** Analyses of immune and stromal cells in different pathological grades, pathological stages within the NA+S and the SA groups.


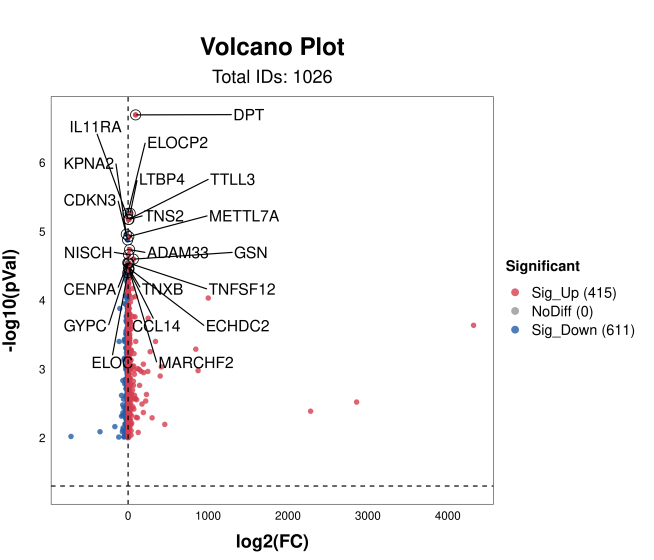


**Figure S4.** Volcano map for intercluster DEGs between the NA+S and the SA groups.


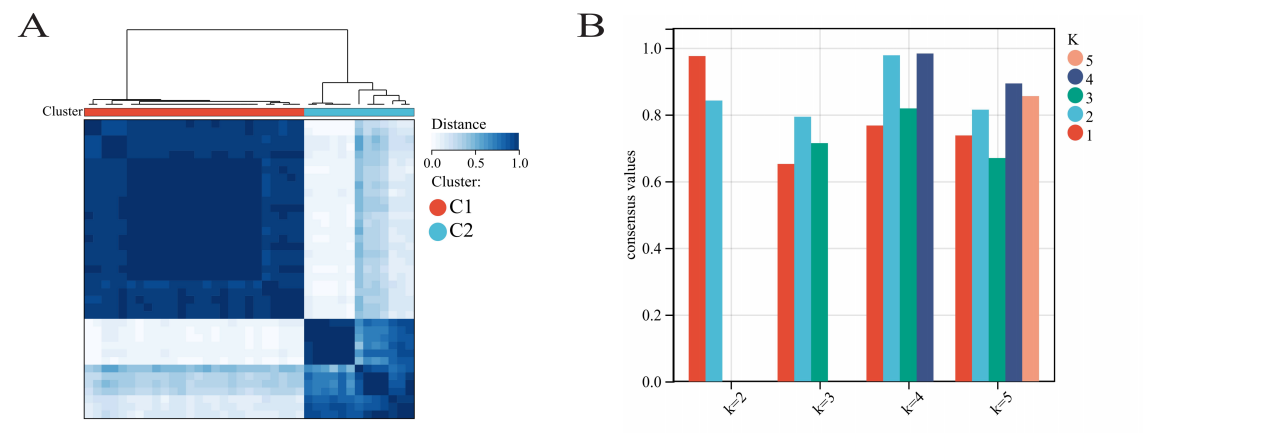


**Figure S5.** Consensus clustering of ICD-related genes in TCGA database.


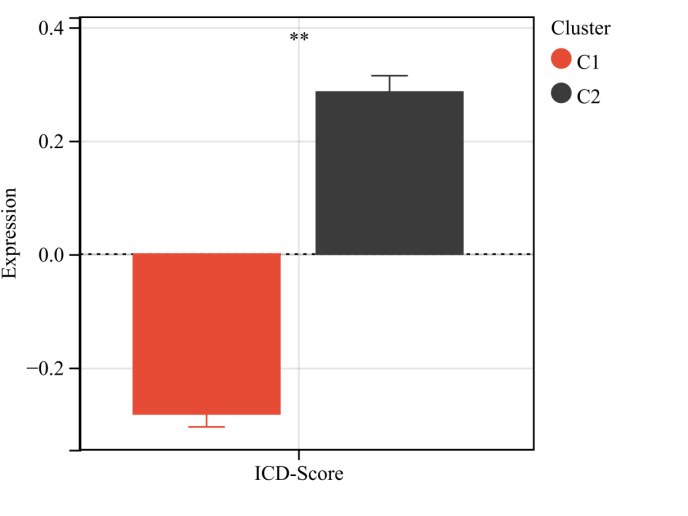


**Figure S6.** C2 was defined as the high-level ICD group.

**
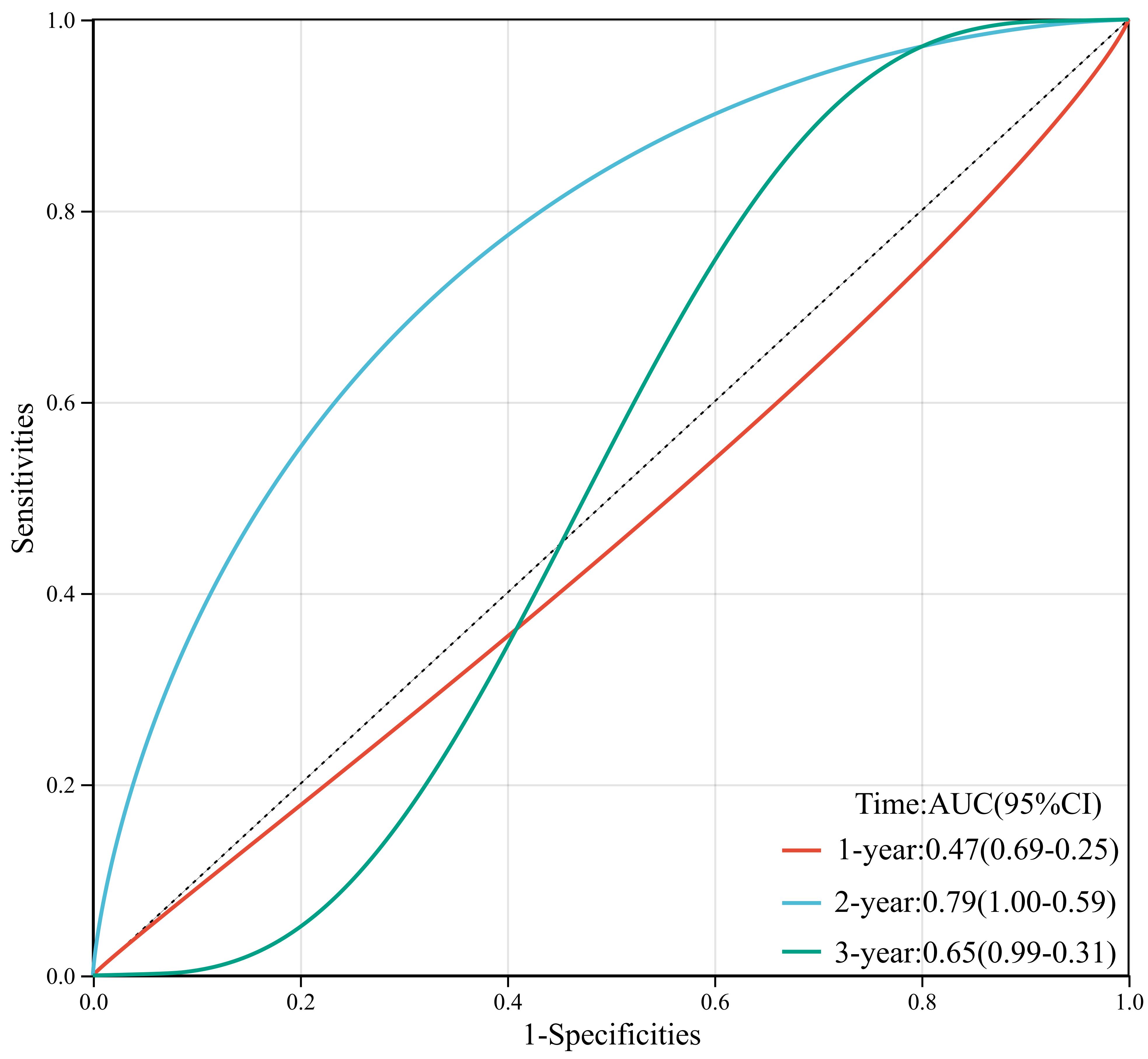
**

**Figure S7.** The predictive ability of our five-gene signature at 1-, 2- and 3-year time points in N+ subgroup.

**Table S1. Demographic characteristics of ESCC patients from TCGA database**

|  | High-Risk (n=32)  No. (%) | Low-Risk (n=62)  No. (%) | Total (n=94) | *P* |
| --- | --- | --- | --- | --- |
| Gender |  |  |  | 0.87 |
| Male | 28(29.7%) | 52(55.3%) | 80 |  |
| Female | 4(4.3%) | 10(10.7%) | 14 |  |
| Age（year） |  |  |  |  |
| Mean±SD | 58.94±11.24 | 58.15±9.87 | 58.41±10.31 |  |
| Median(min-max) | 57 (42,90) | 57.5 (36,84) | 57 (36.00,90.00) |  |
| Pathological stage |  |  |  | 0.14 |
| Ⅰ | 3(3.2%) | 4(4.3%) | 7(7.53%) |  |
| Ⅱ | 14(15.1%) | 41(44.1%) | 55(59.14%) |  |
| Ⅲ | 13(14.0%) | 13(14.0%) | 26(27.96%) |  |
| Ⅳ | 1(1.1%) | 4(4.3%) | 5(5.38%) |  |
| Lymph nodes involvement |  |  |  | 0.51 |
| N0 | 15(16.1%) | 36(38.7%) | 51(54.84%) |  |
| N+(1~any) | 16(17.2%) | 26(28.0%) | 42(45.16%) |  |
| Grade |  |  |  |  |
| 3 | 6(6.4%) | 15(16.0%) | 21(22.34%) | 0.45 |
| 1-2 | 24(25.5%) | 39(41.5%) | 63(67.02%) |  |
| Unknown | 2(2.1%) | 8(8.5%) | 10(10.64%) |  |
| Tumor location |  |  |  | 0.56 |
| Upper-middle | 19(20.2%) | 31(33.0%) | 50(53.19%) |  |
| Lower | 13(13.8%) | 30(32.0%) | 43(45.74%) |  |
| NR | - | 1(1.1%) | 1(1.06%) |  |

The pathological staging of TCGA cohort patients was based on the 6^th^/7^th^ edition of the ESCC-TNM staging criteria issued by Joint American Council on Cancer/International Union for Cancer Control (AJCC/UICC).

**Table S2. ICD-related genes list**

| ATG5 | BAX | CALR | CASP1 | CASP8 | CD4 |
| --- | --- | --- | --- | --- | --- |
| CD8A | CD8B | CXCR3 | EIF2AK3 | ENTPD1 | FOXP3 |
| HMGB1 | HSP90AA1 | IFNA1 | IFNB1 | IFNG | IL10 |
| IL17A | IL17RA | IL1B | IL1R1 | IL6 | LY96 |
| MYD88 | NLRP3 | NT5E | P2RX7 | PDIA3 | PIK3CA |
| PRF1 | TLR4 | TNF |  |  |  |

**Table S3. Primary antibodies list**

| Name | Resource | Cargo available | Company&Nation |
| --- | --- | --- | --- |
| anti-Calreticulin | Rabbit | ab2907 | Abcam, USA |
| anti‐HMGB1 | Rabbit | ab18256 | Abcam, USA |
| anti-SLAMF7 | Rabbit | ab237730 | Abcam, USA |
| anti-IL1R1 | Rabbit | ab229051 | Abcam, USA |
| anti‐beta tublin | Rabbit | ab179511 | Abcam, USA |
| anti‐GAPDH | Rabbit | ab9485 | Abcam, USA |

**Table S4. Primers for qPCR assays**

| Calreticulin | Forward | 5’-AAATGAGAAGAGCCCCGTTCTTCCT-3’ |
| --- | --- | --- |
|  | Reverse | 5’-AAGCCACAGGCCTGAGATTTCATCTG-3’ |
| HMGB1 | Forward | 5’-AAACTTCTCAAGCCCCATCC-3’ |
|  | Reverse | 5’-CAGAAACCAAAGAAATAGCCCC-3’ |
| SLAMF7 | Forward | 5’-AGAGTTTGATCMTGGCTCAG-3’ |
|  | Reverse | 5’-GGTTACCTTGTTACGACTT-3’ |
| IL1R1 | Forward | 5’-GTGCTACTGGGGCTCATTTGT-3’ |
|  | Reverse | 5’-GGAGTAAGAGGACACTTGCGAAT-3’ |

*Primers were produced from Sangon biotech institute (Shanghai, China).


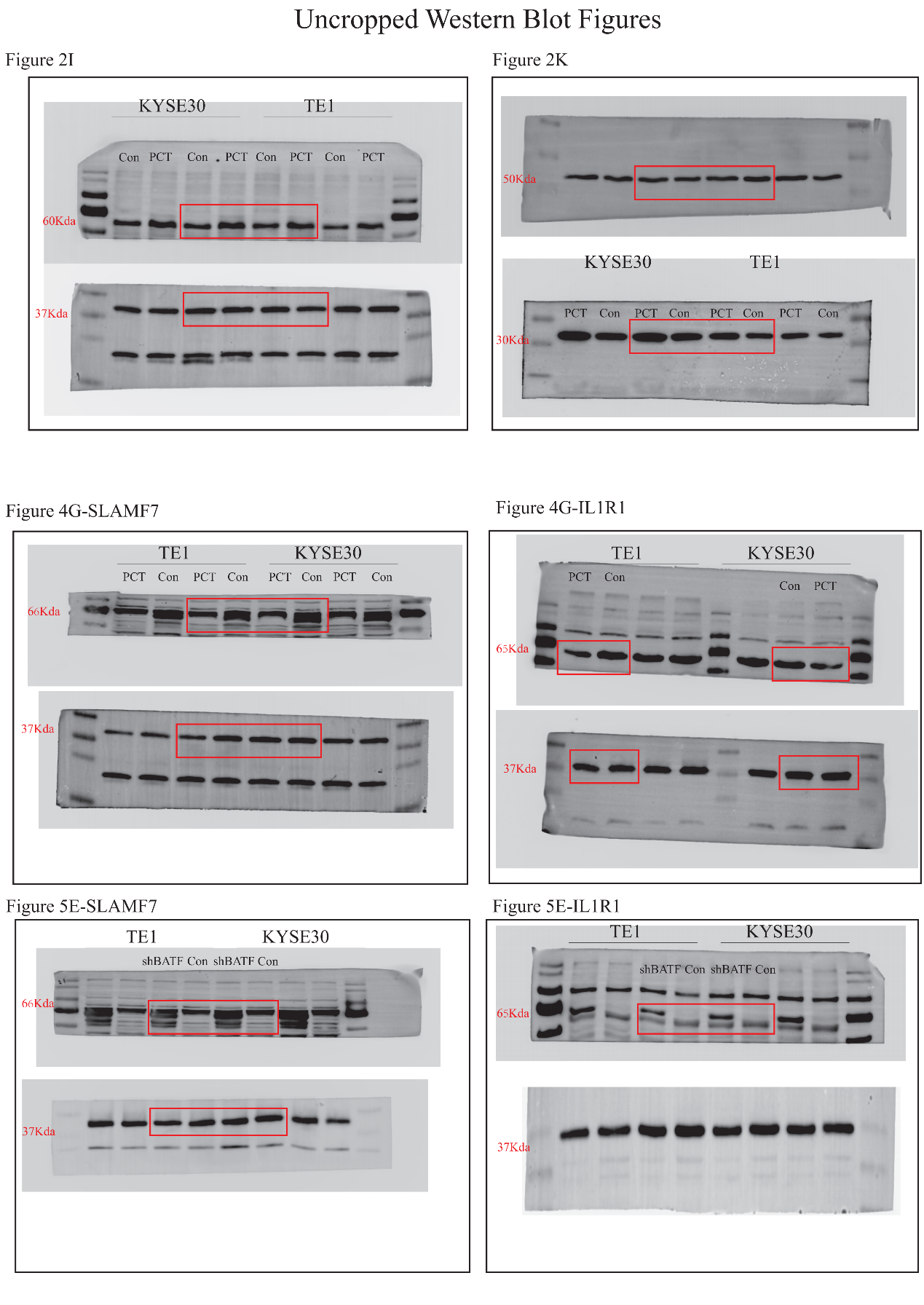

Supplement: Supplementary file 1 — Supporting information [file MCO2-6-e70171-s001.docx]
